# The Work of Hospital Almoners—II

**Published:** 1917-07-21

**Authors:** 


### THE MATRONS' AND SISTERS' DEPARTMENT.

### THE WORK OF HOSPITAL ALMONERS.—II.

### Address by Miss Cummins at the London School of Economics.

THERE are several reasons why the almoner's office acts as a good sorting-house. Firstly, the attitude of the man or woman coming to hospital differs very much from his or her attitude when asking for any other kind of help. And this, I think, is due to the feeling that disease is an ill to which all flesh is heir, and one feels that there is a certain freemasonry among humanity in respect of it.

Then the patients have a real regard for the doctor, and this is a great lever for the almoner.

Then they come to us in a very impressionable condition. They have perhaps faced for the first time the actual bedrock when they heard the doctor's verdict, or they have seen sights among the other patients which have made them realise the terrors of pain, and the closeness of death. I often feel almost frightened by the power of the weapons we seem to handle, and I am absolutely convinced that the visit to the hospital is sometimes a turning-point in a patient's life. They also, I think, find it a certain relief to talk things out. In the first shock they may feel unable to look round at all, are almost crushed, and thankful to listen to anyone who is able to go into the whole circumstances, and plan.

The almoner's duty, then, is to ensure that as far as possible the doctor's instructions are carried out, and moreover to procure any such adjuncts, such as special diet, convalescent letters, surgical appliances, and home nursing, as may be necessary. Linked up with every outside agency, and with the trained knowledge of the working and scope of all charitable societies, it is for her to plan and contrive that the patients have every possible opportunity to profit by the medical treatment, and it is her duty to report when the social circumstances are such that recourse to the Poor Law seems the only possible solution of the problem.

Just now I referred to the patients being visited and watched. The doctor may have ordered complete rest, open windows, special diet, etc., orders that can perhaps be carried out quite simply, but which are constantly ignored by patients who believe that medicine alone will cure them. It is for the almoner, then, to attack the difficulty in the patient's own home. Naturally, she herself cannot do this, but again the spirit of co-operation comes into the work. She applies to an outside organisation to undertake the visiting, to go down to the patients' homes, and in a friendly way educate them and plan with them how best to arrange. If the mother is obliged to rest a leg for varicose veins, the visitor may find a neighbour able to give a hand with the housework; or, if the open window causes a draught, she may be able to help alter the position of the bed; or invalid diet may have been ordered, and she will be called on to advise how to prepare it.

The value of home visiting in connection with the treatment of our patients cannot be over-estimated. We make inquiries into the home circumstances when the patient first attends, not from any wish to pry into their affairs, but simply because it is impossible to help them either by advice, monetary relief, or relief in kind, unless we know how they are really placed at home. Often the inquiry then seems to be sufficient, and they are passed for treatment and watching at the hospital alone, but very often the inquiry does not account for the patient's condition, and one feels that the explanation can only be revealed by a visit to the home.

For instance, the little girl suffering from chorea—there is no history of rheumatism, the mother seems sensible and assures me that she is keeping the child warm and dry and free from excitement, and yet the movements continue. The visitor finds that the house is in a noisy street, and moreover that a lodger comes home drunk on Saturday nights, and makes a great deal of noise, shouting and banging doors, etc. Clearly the little one is not likely to improve, so the physician is seen and possibly admits the child to the wards, and she is then sent to a small convalescent home. But the matter must not rest there, or all the good is likely to be nullified when the child returns. Many other visits must be paid to induce the mother to move, and to see that she understands how to look after the child on her return.

Again, a woman in the wards is not getting on as well as she ought, and it is found that she is worrying about the children; not that the neighbour next door is not cooking the meals regularly, but that the children are possibly not having their hair brushed, and being properly washed and sent clean to school. Possibly, to achieve this, both the husband and neighbour must be seen, and special arrangements are made.

Sometimes you find a man coming backwards and forwards to hospital looking very shabby and ill-nourished and depressed, and yet on paper the family income looks enough to provide decent clothing or food. But a visit to the home finds his wife a careless, untidy woman and a bad manager. She is not a drunkard, but she likes her glass of beer, and wastes much time gossiping at the nearest public-house. Now, I do not mean to say that the visitor can alter all this, but I do say that persistent visiting based on real interest and goodwill can achieve much, and in addition, when seeing the man on his attendance at hospital, it is possible sometimes, without betraying the source of one's knowledge, or leading the man to speak badly of his wife, to help them now and again to understand each other better, and to show him how, if he expects the house clean and tidy, he may himself possibly improve the conditions, or to point out that women generally respond to encouragement.

But, again, the patient may have passed through the doctor's hand, and have quietly acquiesced in all he has said, and then, when talking to the almoner, have disclosed a further history. The almoner listens to the long and involved story and tries to disentangle the real from the imaginary. Many diseases are caused by fear, self-absorption, and remorse, and many patients only come to the hospital for diseases caused by intemperance, ignorance, and alcohol. No lay person, no social worker, could diagnose or treat such patients, but, after the physician has diagnosed and ordered, his valuable time and skill will be to a large extent wasted unless the root of the trouble is bravely tackled.

**(Continued on p. 327.)**

NOTE.—The first article appeared on July 14, p. 299.

### THE WORK OF HOSPITAL ALMONERS.—II. (continued).

I do not claim that all social wrong can be cured. One of the saddest sights in a hospital is the patient who comes too late for the doctor's skill, and the same can be said from the social aspect. But, fortunately, in a large percentage the character of the patient can be treated. After watching the wonderful skill and care of the medical staff, one feels justified in claiming that it is entitled to be augmented by the social worker.

Dr. Cabot, of the Massachusetts General Hospital, in his book on this subject, says :—

"Therefore to make the doctor's work worth while to himself and to the patient, it must be done (in hospitals) in co-operation with someone who has time and ability to teach hygiene and to see that it is carried out, to study the home conditions and report on their part in causing and prolonging disease, and to help modify those conditions, financial, mental, moral, which stand between the patient and recovery.

"This someone is the social worker—a man or woman trained to think of a human being as a whole, just as naturally as the physician concentrates attention upon a part."

After a time the doctors get into the habit of referring cases to the almoner, often asking for a home report before deciding on treatment; or, again, I find some such note as this on the hospital letter : "Has this patient cause for special worry, or can you find out if she has had a shock?" Or, "Can the almoner find out whether this girl has home troubles?" And in turn we often apply to the staff, telling them of the difficulty that besets a patient, and asking help and support in some particular line of action. In America they have termed this team-work, and the team is often a long one, consisting of the doctor, the nurse, the patient, the almoner, and one or more outside agencies.

I will not take up your time in telling of individual cases, but I would like to mention one special class of patients, to whom of all others I feel that the hospital stands in a specially important place. I refer to single girls who are pregnant, or who are suffering from venereal disease. Again it is to Dr. Cabot that I would refer when he suggests that the doctor's verdict in such cases is liable just to push them further down the precipice.

Utterly lonely, feeling degraded and pariahs, they too often give way to despair or recklessness as the hospital door closes behind them. They may, and probably have, feared their condition, but now they know it as a certainty.

Here again it is needless for me to dwell long on such cases. This audience is able to understand and enter into my plea, which is that side by side with the verdict should be some understanding woman, who, while not glossing over the past, should be there with experience and time to face the future and help build up and construct a possible basis for a healthier and better life.

My mind instinctively goes back to a little typist who two or three years ago came into the gynæcological department, and who was handed on by the physician with a note containing the diagnosis and the words "Can you help?" That girl came determined to do away with herself if what she feared proved true, and, as she said afterwards, "I never expected that anyone would have time to understand." We have been in touch with her ever since, and I am able to say that she is a fairly happy, healthy member of the community to-day, and quite independent.

And here again I need not tell you of the many cases whom one seems unable to help, the many instances of failure in one's efforts; nor those cases who go away heedless, to plunge lower; and yet some of them do sometimes remember and come back to the hospital as a place where there is someone "with time to understand."

I will not weary you with too many details of the social work that is undertaken in an almoner's office in regard to the various other departments. It is bound to vary greatly in the different hospitals, and depends also on the existing agencies working within the hospitals' area. The almoner should act solely as the connecting link whenever outside agencies exist to whom she can refer for help, and in this capacity her work must be well known to any here who have worked in relief societies. In the hospital she stands for the social side of the patients' lives, and her dealings with the staff and patients are solely based on this. Amongst outside agencies she represents the medical side in so far as she interprets the needs of the patients and the directions of the doctors. Having no medical training, the term sometimes used, especially in the States, "medico-social worker," seems to me unfortunate; as it would imply a knowledge of medicine which the almoner does not possess or require. It is true that the training of an almoner includes courses in hygiene and physiology, and training in the home treatment of tuberculosis, child welfare, etc., but such training is only given to ensure that the almoner can intelligently understand the directions of the doctor.

### Maternity Work.

Possibly the maternity work may be worth a brief summary. As a rule a general hospital serves a given area, and sends out doctors for the confinements. Years ago, when I first started as an almoner in South London, the condition of the maternity patients was often appalling, and one wonders how the students carried on the work as they did; for it was no unusual thing for the hospital doctor to be called to a confinement where absolutely no preparations had been made, where there were no sheets, sometimes no bedclothes, no hot water or firing, and only perhaps a leaking jug or saucepan in which to wash the baby, only a drunken neighbour in attendance on the patient, who was herself not quite sober. It took some time to alter these conditions, and doubtless the general improvement in the standard of living has played its part in addition to our own efforts.

Now, when the women apply for their cards for the maternity ward, or for a doctor to attend them in their homes, the almoner goes thoroughly into each case. She learns as much as she can of their special circumstances, goes into the question of diet and general instructions, and teaches them the preparations that the hospital expects both for mother and baby—long-sleeved vests, cradles, etc., and the actual requirements for the period of confinement. A worker is then sent to the home. If the preparations are inadequate, the cards are withheld to give the parents time to prepare. Before the pressure of the war one of the obstetric house-surgeons kindly gave up an evening a week to interviewing difficult fathers for us, as we realised that it was unfair to put all the pressure on the woman. These interviews were of great constructive value. In very bad cases where the parents

(Concluded on page 326, column 2.)

### The Work of Hospital Almoners—II.

(*Continued from* p. 327).

refuse to make suitable provision the cards are refused, and this has led to a wonderful improvement in the standard in our district.

Sometimes poverty prevents suitable preparations, and there help is given by the Samaritan Fund in co-operation with a local relief agency. Great stress is laid during the ante-natal period on breast-feeding, and our mothers are encouraged to feel a pride in being good nursing mothers. If possible, a visitor is attached to each case, who continues during the first year, and the women come up to the hospital if any difficulty arises, or drop us a postcard asking for an immediate visit.

We have baby clinics at the hospital, and the doctor's instructions form the basis of all the visitor's watching and care. The most striking feature has been the wonderful increase in breast-fed babies, and the pride that the parents both take. The weight-card is a very important document to exhibit in the homes. The almoner has naturally the charge of the home conditions in the maternity department, and works in co-operation with the nurses and doctors in the clinics. In a way this work bears very directly on the medical school, as the house men are encouraged to report all home difficulties, and thus at the beginning of their career are brought into contact with social work and its bearings.

Whenever there is a School for Mothers in the district in which a mother attends, the mother and baby are placed under its care, as in no way should the hospital work overlap with such agencies, although it would seem wise for any hospital with a medical school to undertake to be responsible for some small district to which it would act as an infant centre, because infant care is bound to play a very important part henceforward, and it seems advisable that medical students should have full opportunities of studying normal babies during their hospital training.

